# 1-(2-Hydroxy-5-methylphenyl)-3-phenyl-1,3-propanedione Induces G1 Cell Cycle Arrest and Autophagy in HeLa Cervical Cancer Cells

**DOI:** 10.3390/ijms17081274

**Published:** 2016-08-05

**Authors:** Jie-Heng Tsai, Li-Sung Hsu, Hsiu-Chen Huang, Chih-Li Lin, Min-Hsiung Pan, Hui-Mei Hong, Wei-Jen Chen

**Affiliations:** 1 Institute of Biochemistry, Microbiology and Immunology, Chung Shan Medical University, Taichung 402, Taiwan; a19851102@hotmail.com (J.-H.T.); lsh316@csmu.edu.tw (L.-S.H.); 2 Clinical Laboratory, Chung Shan Medical University Hospital, Taichung 402, Taiwan; 3 Department of Applied Science, National Hsinchu University of Education, Hsinchu 300, Taiwan; jane@mail.nhcue.edu.tw; 4 Institute of Medicine, Chung Shan Medical University, Taichung 402, Taiwan; dll@csmu.edu.tw; 5 Department of Medical Research, Chung Shan Medical University Hospital, Taichung 402, Taiwan; hhm@csmu.edu.tw; 6 Institute of Food Science and Technology, National Taiwan University, Taipei 106, Taiwan; mhpan@ntu.edu.tw; 7 Department of Biomedical Sciences, Chung Shan Medical University, Taichung 402, Taiwan

**Keywords:** 1-(2-hydroxy-5-methylphenyl)-3-phenyl-1,3-propanedione, G1 arrest, autophagy, light chain 3 (LC3), Beclin-1, p62

## Abstract

The natural agent, 1-(2-hydroxy-5-methylphenyl)-3-phenyl-1,3-propanedione (HMDB), has been reported to have growth inhibitory effects on several human cancer cells. However, the role of HMDB in cervical cancer remains unclear. Herein, we found that HMDB dose- and time-dependently inhibited growth of HeLa cervical cancer cells, accompanied with G1 cell cycle arrest. HMDB decreased protein expression of cyclins D1/D3/E and cyclin-dependent kinases (CDKs) 2/4/6 and reciprocally increased mRNA and protein levels of CDK inhibitors (p15, p16, p21, and p27), thereby leading to the accumulation of hypophosphorylated retinoblastoma (Rb) protein. HMDB also triggered the accumulation of acidic vesicles and formation of microtubule-associated protein-light chain 3 (LC3), followed by increased expression of LC3 and Beclin-1 and decreased expression of p62, suggesting that HMDB triggered autophagy in HeLa cells. Meanwhile, suppression of the expression of survivin and Bcl-2 implied that HMDB-induced autophagy is tightly linked to apoptosis. Exploring the action mechanism, HMDB induced autophagy via the modulation of AMP-activated protein kinase (AMPK) and mTOR signaling pathway rather than the class III phosphatidylinositol 3-kinase pathway. These results suggest that HMDB inhibits HeLa cell growth by eliciting a G1 arrest through modulation of G1 cell cycle regulators and by concomitantly inducing autophagy through the mediation of AMPK-mTOR and Akt-mTOR pathways, and may be a promising antitumor agent against cervical cancer.

## 1. Introduction

Cervical cancer is one of the leading causes of gynecologic cancer death in women worldwide and approximately 500,000 new cervical cancer cases are deduced, contributing to 280,000 deaths each year [1]. More than 80% of cervical cancer patients are diagnosed in developing countries [2], and new cases suffering from cervical cancer are approximately 150,000 in China per year, accounting for about 30% of new cases worldwide. More than 99.7% of cervical cancer cases contain one or more of the oncogenic human papillomavirus (HPV) genotypes that cause cervical cancer [3]. HPV infection has been considered as the most key factor responsible for the development of cervical cancer; especially, HPV 16, 18, 31, and 33 infections are characterized as the primary risk factors highly associated with cervical cancer. Among them, HPV-16 and -18 infections account for about 70% of cervical cancer cases [4]. Two primary HPV viral oncoproteins, E6 and E7, are required for the development of cervical cancer with the transformed phenotypes. For example, E6 protein induces p53 degradation by the ubiquitin-proteasome mediated pathway. E7 protein interacts with retinoblastoma (Rb) protein and preventing Rb binding to cell cycle-related transcription factor E2F [5,6], which give rise to the loss of Rb/E2F complexes, the release of E2F, and the subsequent progression of cell cycle from G1 to S phase [7,8].

Abnormal regulation of cell cycle is a result of cancer development [9]. In mammals, cell cycle progression is strictly for the regulation of a set of proteins, including cyclin-dependent kinases (CDKs), cyclins, and CDK inhibitors (CKIs) that control cell cycle progression at G1, S, and G2/M checkpoints [10]. In early stage of G1 phase, the D-type cyclins emerge and accumulate in the nucleus due to mitogenic signal firing, and then forms cyclin-CDK4/6 complexes, resulting in the initial phosphorylation of Rb proteins. In late stage of G1 phase, the cyclin E/CDK2 complex further promotes the formation of highly phosphorylated Rb protein, thus releasing E2F and eventually leading to the entry of the S phase. The mechanism how to regulate the activation of CDKs is well-established. Generally, the activity of CDKs can be mediated by altering their phosphorylation status on a conserved threonine residue or by interacting with CKIs [11]. CKIs consist of two families, including the Ink4 and the Cip/Kip families [12]. The p15, p16, p18, and p19 proteins which belong to the Ink4b family bind to CDK4/6 to prevent the formation of CDK4/6-cyclin complexes. The high protein levels of the Cip/Kip family (such as p21, p27, and p57) inactivate CDK2 activity, most probably by leading to the stoichiometry in the CDK2-cycle E complexes [12]. In addition, CDK inhibitors induce autophagy in cancer-associated fibroblasts and cancer cells [13,14].

Autophagy, a process for major intracellular degradation, occurs when cells undergo stress conditions, such as nutrient starvation, exposure of radiation or cytotoxic compounds, or suffering from cancer, to promote cell survival or to result in type II programmed cell death [15]. During autophagy, the cytoplasm components or organelles for determined degradation are conveyed to the double-membrane vesicle, known as autophagosome, and then acidified for maturation to pass into acidic vesicular organelles (AVOs) [16]. Eventually, the AVOs fused with lysosomes to form autophagolysosomes which digest their internal components by lysosomal hydrolases [17,18]. Beclin-1 and microtubule-associated protein 1A/1B-light chain 3 (LC3), two hallmarks of autophagy, regulate the beginning of mammalian autophagy [19]. Beclin-1 plays a role in involving in the signaling pathway required for the induction of autophagy and in the onset of autophagosome formation [19,20]. The overexpression of Beclin-1 inhibits the proliferation and growth of HeLa cells in vitro and in vivo, while inducing autophagy and subsequent apoptosis of HeLa cells [21]. LC3 consists of a soluble form, LC3I, and a lapidated form, known as LC3II. The presence of LC3II is directly linked to autophagy, because LCII is recruited in the formation of autophagosomes. The cellular stress triggers LC3I conjugated to phosphatidylethanolamine to constitute the lapidated LC3II, which is a component of autophagosomes and so far conceived as a marker of autophagy [20,22]. Compared to normal cervical epithelial tissues and cervical squamous carcinoma tissues, Beclin-1 is highly expressed in 96.2% (25/26) versus 28.0% (14/50) of cervical cancer patients, and LC3 is highly expressed in 76.9% (20/26) and 26.0% (13/50) of cervical cancer patients, respectively [23], suggesting the induction of autophagy may be an accessible tactic for cervical cancer therapy.

1-(2-Hydroxy-5-methylphenyl)-3-phenyl-1,3-propanedione (HMDB) is a β-diketone structural compound that has growth inhibitory effects on several human cancer cells [24,25]. HMDB was suggested to function as an anticancer drug via modulating the mitochondrial functions that are regulated by reactive oxygen species, upregulating CCAAT/enhancer binding protein delta (CEBPD), growth arrest DNA damage-inducible gene 153 (GADD153), BAD, and p21, and downregulating BCL2L1 (BCL-XL) [26]. However, the growth-inhibitory and autophagy-inducing effects of HMDB on cervical cancer have not yet been elucidated. To this end, the HeLa cervical cancer cell line was employed as an in vitro model to explore the anti-cancer effect of HMDB, focusing on the induction of autophagy and the resultant growth inhibition. Moreover, the effects of HMDB on the activation of several kinases and the following signaling pathways critically responsible for cell autophagy were explored. The present study may provide novel evidence that HMDB may be a potent cancer chemopreventive agent against some types of cervical carcinomas.

## 2. Results

### 2.1. Inhibition of Cell Growth and Cell Cycle Progression at G1 Phase of HeLa Cells by 1-(2-Hydroxy-5-methylphenyl)-3-phenyl-1,3-propanedione (HMDB)

**Figure 1:**
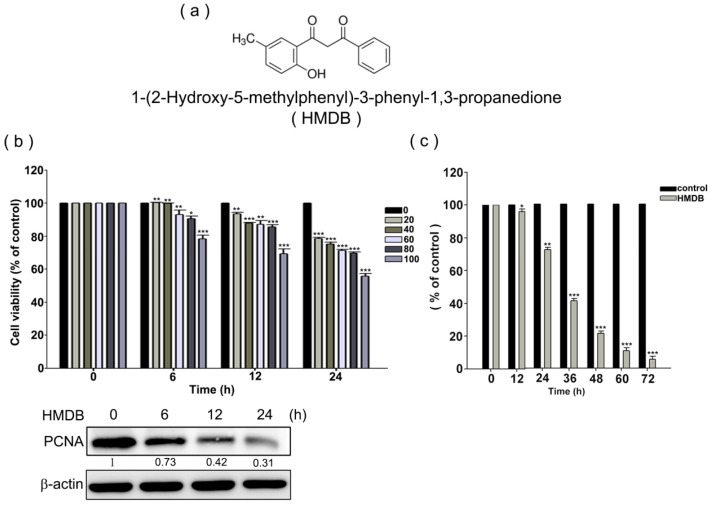

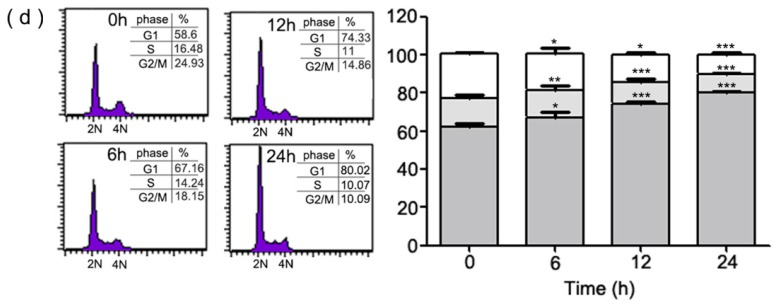
HMDB inhibited proliferation of HeLa cells via inducing the G1 cell cycle arrest. (**a**) The chemical structure of HMDB; and (**b**) the effect of HMDB on cell viability of HeLa cells. Cells were treated with a variety of dosages of HMDB for 0–24 h or (**c**) with 40 µM HMDB for different time periods. Cell survival was determined using 3-(4,5-dimethylthiazol-2-yl)-2,5-diphenyltetrazolium bromide (MTT) assay and trypan blue exclusion assays, respectively. The protein levels of PCNA were determined by Western blotting; and (**d**) a histogram of the cell cycle distribution. HeLa cells were treated with 40 μM HMDB for 0, 6, 12, and 24 h. Cell distribution at G1, S and G2/M phase was determined using flow cytometry. All of the data resulted from repeating independent experiments three times and results are expressed as mean ± SE. Values were statistically significant (versus HMDB treatment) for * *p* < 0.05, ** *p* < 0.01, *** *p* < 0.001 as compared with the control group.

To investigate the growth inhibitory activity of HMDB (Figure 1a), we first examined the growth-inhibitory effect of HMDB on HeLa cells using 3-(4,5-dimethylthiazol-2-yl)-2,5-diphenyltetrazolium bromide) (MTT) and trypan blue exclusion assays, respectively. Human HeLa cervical cancer cells were treated with various concentrations of HMDB at the indicated time points. As shown in Figure 1b,c, HeLa cell growth was inhibited by HMDB in a dose- and time-dependent manner. HMDB also time-dependently reduced the expression of nuclear antigen proliferating cell nuclear antigen (PCNA), a hallmark of proliferation expressed in proliferating cells. Next, to determine whether HMDB has an effect on cell cycle, we analyzed the effect of HMDB on the cell cycle distribution by flow cytometry using PI staining. As shown in Figure 1d, HMDB at 40 μM caused G1 cell cycle arrest in a time-dependent manner. In particular, approximately 58.6% of the untreated HeLa cells were in the G1 phase, while the cells exposed to 40 μM HMDB exhibited a considerably greater proportion of G1 cells (approximately 80.02%). The increased number of cells in the G1 phase after HMDB treatment was tightly associated with the decreased number of cells in the S and G2/M phases compared to the control.

### 2.2. Modulation of the Expression of G1 Cell Cycle Checkpoint Regulators by HMDB in HeLa Cells

**Figure 2:**
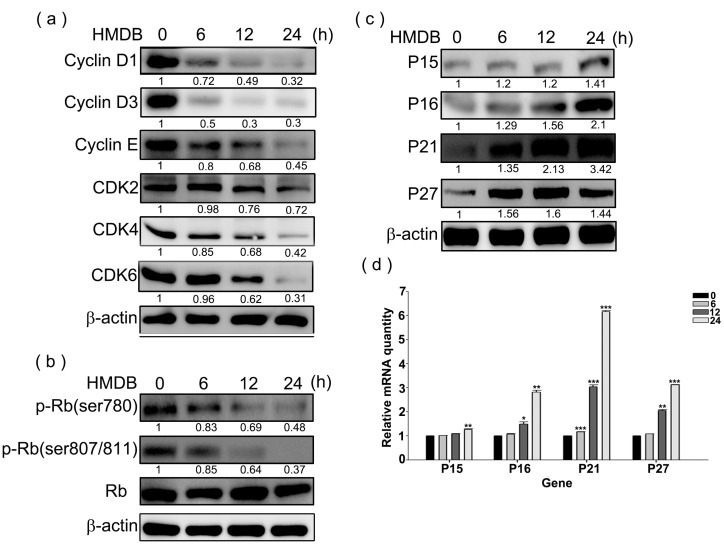
Effects of HMDB on the expression of G1-related cyclins, cyclin-dependent kinases (CDKs), and CDK inhibitors (CKIs). (**a**) Relative protein expression levels of cyclin D1/D3/E, and CDK4/6/2 expressed in the G1 phase; (**b**) the total and phosphorylated forms of retinoblastoma (Rb) with specific antibodies for each; and (**c**) the change in the protein expression levels of CKIs (p15, p16, p21, and p27). HeLa cells were exposed to 40 μM HMDB for the indicated times. Then, cellular extracts were harvested and the protein levels were visualized by Western blotting using antibodies against G1 cell cycle regulators as indicated. The β-actin acts as an internal control for evaluating protein loading; and (**d**) the changes in mRNA expression levels of CKIs, including p15, p16, p21, and p27, by HMDB. The relative amounts of target mRNA, collected from HMDB-treated HeLa cells, were determined by qRT-PCR for the indicated time. All of the results that come from independent experiments three times are expressed as mean ± SE. The relative amounts of protein levels on the Western blots were quantitated with a computerized densitometer (ImageQuant LAS4000 Digital System, GE Healthcare, Uppsala, Sweden) compared to the control group. Values were statistically significant for * *p* < 0.05, ** *p* < 0.01, *** *p* < 0.001 as compared with the control group (without HMDB treatment).

Given that HMDB induces G1 cell cycle arrest in HeLa cells, we investigated whether HMDB treatment changes the expression profile of cell cycle regulatory proteins such as cyclin D, cyclin E, and their associated CDK4/6 and CDK2, required for G1 to S transition in cell cycle. HeLa cells were treated with 40 μM HMDB for the indicated time points and then cell extracts were harvested for Western blotting. As demonstrated in Figure 2a, HMDB distinctly reduced the protein expression of cyclin D1/D3/E, and CDK4/6/2 in a time-dependent manner. These results indicate that inhibition of the expression of G1 phase-related cyclins and CDKs might be a critical event in the HMDB-mediated growth arrest in HeLa cells.

The phosphorylation of the Rb protein mediated by G1-related cyclin/CDK complexes is necessary for cell cycle progression from G1 to S phase. To assess whether the down-regulation of the expression of cyclins and CDKs by HMDB can lead to the dephosphorylation of the Rb protein, the phosphorylation status of the Rb protein was determined by Western blotting using specific antibodies against the phosphorylated Rb protein after exposure of exponentially-growing HeLa cells to HMDB. As illustrated in Figure 2b, the Rb phosphorylation at Ser780, 807, and 811, associated with the regulation of G1 cell cycle progression were time-dependent inhibited by HMDB from 6–24 h treatment, paralleled with a decrease in the protein levels of cyclin D1/D3/E and CDK4/6. These findings provide evidence that HMDB induces cell cycle arrest at G1 phase via downregulating the expression of cyclins (D1, D3, and E) and CDKs (CDK4 and CDK6).

CKIs are well characterized to prevent the progression of cell cycle from binding and inactivating CDKs alone or cyclin/CDK complexes. To assess the effect of HMDB on the expression of CKIs, we incubated HeLa cells with 40 μM HMDB for the indicated times and then examined determined the protein and mRNA expression levels of CKIs (p15, p16, p21, and p27) by Western blotting and qPCR, respectively. As shown in Figure 2c,d, HMDB clearly resulted in the increase in both protein and mRNA expression of all these CKIs in a time-dependent manner. These results indicate that HMDB may cause the induction of steady-state levels of these CKIs by regulating the transcription of these proteins.

### 2.3. Induction of Cytoplasmic Vacuolation, Formation of Autolysosomes, and Accumulation of Acidic Vesicles in HMDB-Treated HeLa Cells

**Figure 3:**
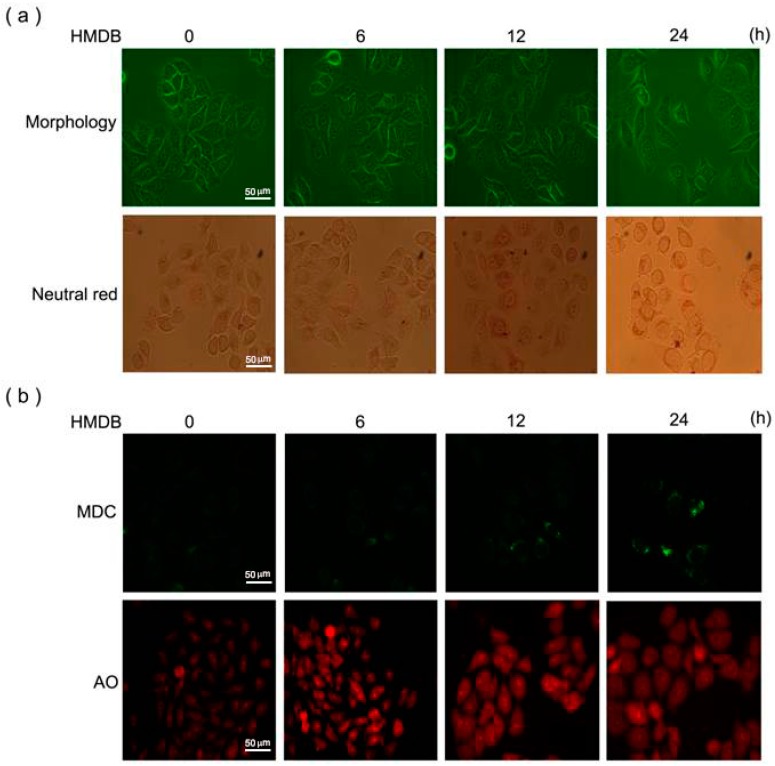
HMDB increased the number of massive vacuoles with acid content and the accumulation of autolysosomes in HeLa cells. (**a**) The cells were treated with 40 μM HMDB for the indicated times. Morphological changes and representative photographs of HeLa cells after neutral red staining in response to HMDB were observed by light contrast microscopy; (**b**) microphotograph of cells stained with monodansylcadaverine (MDC) and acridine orange (AO). Scale bar, 50 µm.

As shown in Figure 3a, upper panel, we observed that HMDB induced a time-dependent increase in the formation of intracellular vacuoles in HeLa cells. In addition, we found that the vacuolar content was acidic through a neutral red staining shown in the lower panel, suggesting the presence of lysosomal content. Combined with the growth inhibition by HMDB, we suggested that HMDB-induced vacuolization in HeLa cells may be autophagic. To demonstrate that these acidic vesicles induced by HMDB are linked to autophagy, the formation of autolysosomes was detected by monodansylcadaverine (MDC) and acridine orange (AO) staining, respectively, which are two remarkable signs of autophagy. As shown in Figure 3b, HMDB elicited a time-dependent increase in MDC- and AO-stained cells, respectively; suggesting autophagy-mediated cell death may be, at least partly, involved in the action mechanism of HMDB-induced growth inhibition in HeLa cells.

### 2.4. Formation of Autophagic Vacuoles with the Increases in Microtubule-Associated Protein 1 Light Chain 3 (LC3) and Beclin-1 Expression Induced by HMDB

**Figure 4:**
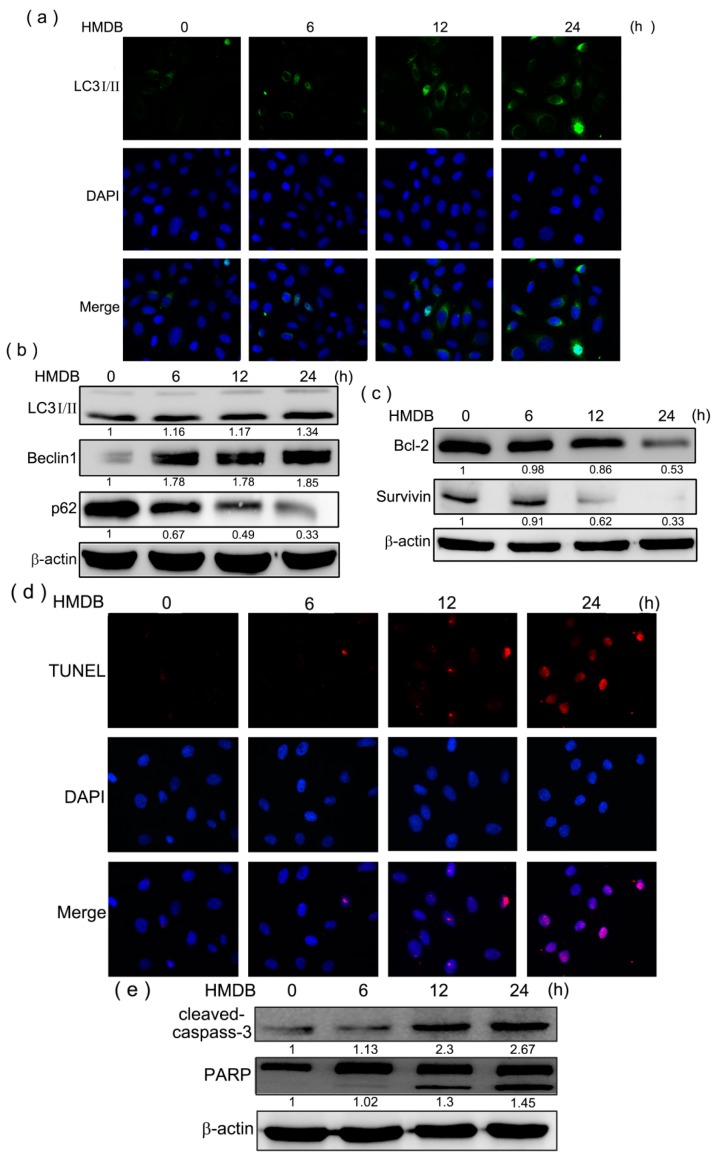
HMDB induced autophagy and apoptosis in HeLa cells. (**a**) HeLa cells were treated with 40 μM HMDB for the indicated times, fixed and incubated with rabbit anti-LC3-II primary antibody. After incubation with Alexa Fluor 488 phalloidin (green) for conjugated anti-rabbit secondary antibodies, immune-labeled cells were monitored by microscopy; (**b**) HeLa cells were treated with 40 μM HMDB and then the protein expression levels of LC3-II, Beclin-1, and p62 were determined by Western blotting for the indicated times; (**c**) the cells were treated with 40 μM HMDB for different times. Cells were harvested and lysed for the detection of the indicated protein expression by Western blotting; (**d**) the cells were treated with 40 μM HMDB for the indicated times, and then the apoptotic cells were examined by TUNEL assay; and (**e**) the apoptosis-related proteins, cleaved caspase-3, and poly (ADP-ribose) polymerase (PARP), were assessed using Western blotting. The densities of the band on the Western blots from three independent experiments were calculated using a computerized densitometer (ImageQuant LAS4000 Digital System, GE Healthcare, Uppsala, Sweden). Nuclei were stained with 4′,6-diamidino-2-phenylindole (DAPI).

Immunofluorescence staining disclosed that HMDB-treated HeLa cells accommodated the acquisition of numerous large autophagic vacuoles in the cytoplasm. To further ascertain that HMDB may induce autophagy in HeLa cells, we assessed the expression and distribution of LC3-II, a hallmark of autophagy present in the autophagosomal membrane. The results showed that HMDB resulted in a time-dependent increase in LC3-II expression in the cytoplasm of HeLa cells (Figure 4a). The protein levels of LC3-II, Beclin-1 (an autophagic mediator promoting the nucleation of the autophagosomal membrane) and p62 expressed during the early stage of autophagy were also monitored by Western blotting. As shown in Figure 4b, HMDB increased the expression of autophagosome-associated LC3-II and the primary pro-autophagic protein Beclin-1 in HeLa cells in a time-dependent manner after 40 μM HMDB treatment. Meanwhile, the levels of p62 protein, a maker of autophagic degradation, were decreased in response to HMDB treatment. Furthermore, the expression levels of B-cell lymphoma 2 (Bcl-2, an inhibitor of Beclin-1 as well as an anti-apoptotic factor) and survivin (a molecule that inhibits autophagy-dependent apoptosis) were determined by Western blotting. Incubation of HeLa cells with HMDB at different time intervals revealed that HMDB led to decreases in the expression levels of Bcl-2 and survivin (Figure 4c). These results suggest that HMDB-induced autophagy plays a suppressive role in HeLa cell survival through apoptosis. Then, we assess whether HMDB induces HeLa cells undergoing apoptosis, apoptosis was determined by the terminal deoxynucleotidyl transferase dUTP nick end labeling (TUNEL) assay. The results showed that HMDB resulted in a sustained increase in apoptotic cells in a dose-dependent manner (Figure 4d). Furthermore, HMDB dose-dependently increased cleaved forms of caspase-3 and Poly (ADP-ribose) polymerase (PARP), the hallmarks of apoptosis (Figure 4e). Collectively, these results provide evidence that HMDB-induced autophagy is tightly linked to apoptosis.

### 2.5. HMDB-Induced Autophagy Is Linked to the Mediation of AMPK/mTOR and Akt/mTOR Signaling

**Figure 5:**
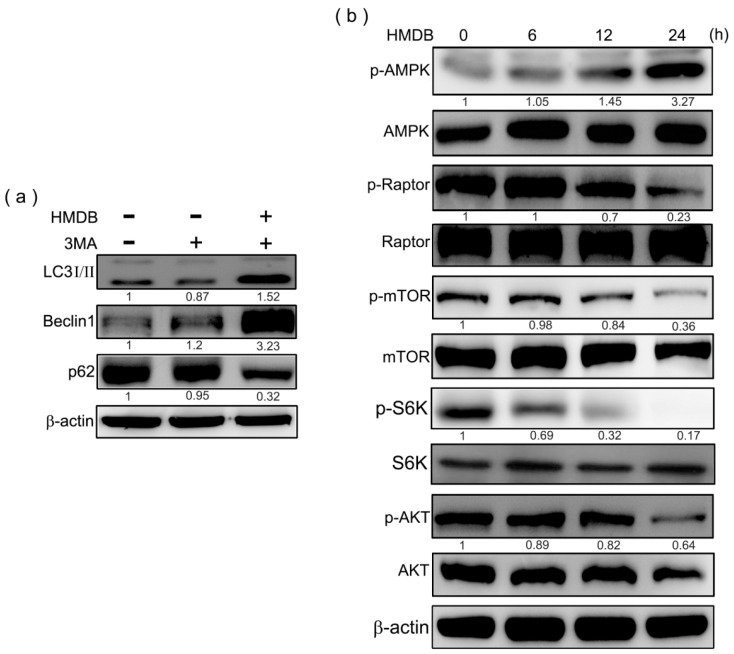
Modulation of class III PI-3K and AMPK/Akt/mTOR signaling was linked to HMDB-induced cell cycle arrest and autophagy in HeLa cells. (**a**) The cells were pretreated with 1 mM autophagy inhibitor, 3-methyladenine, followed by 40 μM HMDB treatment for 24 h. The expression of the indicated proteins was determined by Western blotting; (**b**) HeLa cells were incubated in the presence of 40 μM HMDB for various time points. Cell extracts were harvested for determining the indicated protein expression by Western blotting. The densities of the band on the Western blots from three independent experiments were calculated using a computerized densitometer (ImageQuant LAS4000 Digital System).

The class III phosphatidylinositol 3-kinase (PI-3K), which complexes with Beclin-1 is necessary for the initiation of autophagy. Next, we used the class III PI-3K and autophagy inhibitor, 3-methyladenine, to examine the detailed mechanism by which HMDB induces autophagy. HeLa cells were pretreated with 1 mM 3MA for 1 h, followed by exposure to 40 μM HMDB for 24 h. Then the cell lysates were applied to Western blotting to monitor the protein expression of LC3-II, Beclin-1, and p62. As shown in Figure 5a, pretreatment with 3MA decreased the protein levels of Beclin-1 and LC3-II, and recovered p62 protein levels in HeLa cells; however, 3MA cannot prevent the elevation of Beclin-1 and LC3-II expression levels induced by HMDB in HeLa cells.

The interplay of AMPK (a sensor and positive regulator of autophagy) and the mTOR pathway have been well-known to regulate the occurrence of autophagy. AMPK can phosphorylate Raptor (regulatory associated protein of mTOR), an essential component of mTOR complex 1 (mTORC1), the activity of which blocks autophagy or can phosphorylate TSC2 (tuberous sclerosis complex 2) that directly inhibits Ras homolog enriched in brain (Rheb)-mediated mTORC1activation. To evaluate the signaling pathways responsible for the induction of HMDB-mediated autophagy, we assessed the activation statuses of the main regulators involved in the mTOR signaling pathway in HeLa cells. HMDB triggered the phosphorylation/activation of AMPK reciprocally accompanied with the downregulation of the phosphorylation statuses of Raptor, mTOR and S6K (mTOR downstream substrate) in a time-related manner. HMDB also had an inhibitory effect on the phosphorylation of Akt that negatively regulate TSC2 to release its inhibition on Rheb (Figure 5b). These results suggested that the activation of AMPK and inhibitory modification of Akt-mTORC-S6K signaling axis by HMDB may contribute to autophagic induction and growth inhibition in HeLa cells.

## 3. Discussion

Cervical cancer is developed from malignant cells that form in the cervix. Although a majority of cervical cancer patients have benefited from neoadjuvant chemotherapy together with concurrent chemotherapy and radiotherapy, the survival rate remains poor in cervical cancer patients with relapse or recurrence [27]. Resistance to chemotherapy is one of the common causes of treatment failure in patients suffering from cervical cancer [28,29]. HMDB has been proven to inhibit cell growth in various human cancer cells [25]. Here, we demonstrate that HMDB inhibits proliferation through inducing G1 cell cycle arrest and autophagy in HeLa cervical cancer cells. The results clearly indicate that HMDB induced a G1 cell cycle arrest through downregulating the expression of cyclin D1/D3/E and CDK2/4/6 and subsequently resulting in the hypophosphorylation of Rb protein. Meanwhile, the protein and mRNA levels of CKIs, including p15, p16, p21, and p27, were upregulated by HMDB treatment. These results suggest that the growth-inhibitory effect of HMDB might stem from the blockade of the G1 to S phase transition in HeLa cells through mediating the expression of CKIs binding to their relative cyclin/CDK complexes, a crucial event for inactivating the activity of cyclin/CDK complexes and restricting the progression of cell cycle [30]. Rb not only controls the G1 to S transition in the cell cycle, but also plays a critical role during cellular senescence in response to cancer therapeutics such as CDK inhibitors [31]. In addition, autophagy is an effector mechanism of senescence [32]. Given that HMDB retained the phosphorylation status of Rb in a low level and induced the occurrence of autophagy in HeLa cells, the growth inhibition of HeLa cells by HMDB might be involved to be a link between Rb-mediated autophagy and senescence followed by the consequential tumor suppression; however, this issue requires further research.

Autophagy inhibits the growth of certain cancer cells [33]. In this study, we found that HMDB increased the formation of intracellular vesicles as well resulted in growth inhibition, suggesting that these presented vesicles are autophagic. By means of neutral red staining, HMDB-induced intracellular vesicles were composed of acidic content, presumably coming from lysosome. In additional, lysosome aggregation was present in HDMB-treated HeLa cells by MDC and AO staining. These results suggest that HDMB treatment promotes the combination of autophagosomes and acidic lysosomes arising at the late stage of autophagy. Moreover, our data provide additional evidence that HMDB-induced autophagosome formation is tightly linked to the modulation of protein expression of Beclin-1, LC3-II, and p62/SQSTM1. During autophagy, Beclin-1 and LC3-II are localized on the membrane of autophagosome accompanied with the downregulation of p62/SQSTM1 [34]. Beclin-1 has repeatedly been reported as a target for applied therapies because its low expression may be attributed to the development of human cancer. Inactivation of Beclin-1 was reported to enhance tumorigenesis in mice [35]. In cervical cancer, Beclin-1 expression was significantly decreased in samples of malignant cervical cancer tissues compared to that in normal or cervical intraepithelial neoplasia tissues [36]. Moreover, positive expression of Beclin-1 in human cervical carcinoma has benefits for patients, resulting in a better prognosis [37]. The proautophagic function of Beclin-1 can be negatively regulated by Bcl-2, another well-known anti-apoptosis factor. Beclin-1 contains a BH3 motif required to bind Bcl-2, Bcl-XL, and Mcl-1, and Bcl-2 binds to Beclin-1 from its BC groove. By interaction with Beclin-1, Bcl-2 can block Beclin-1 interacting with class III PI-3K and decrease class III PI-3K activity, thereby negatively regulating autophagy [38]. Although HMDB had no effect on class III PI-3K-mediated LC-3II expression, it showed a time-dependent inhibition on Bcl-2 expression that may release the pro-autophagic activity of Beclin-1. Meanwhile, downregulation of Bcl-2 by HMDB raises a possibility that HMDB could trigger the induction of autophagy-dependent apoptosis in HeLa cells. To this end, the effect of HMDB on survivin expression was investigated. Survivin has been reported to be overexpressed in cervical cancer and participates in the development and progression of cervical cancer by inhibiting autophagy-dependent apoptosis. Evidence that HMDB suppressed the expression of survivin as well as that of Bcl-2 in a time-dependent manner in HeLa cells (Figure 4c), accompanied with the increased number of TUNEL-positive cells and cleaved forms of caspase-3 and PARP (Figure 4d,e) suggested HMDB might at least partly inhibit cell growth of HeLa cells via inducing the autophagy-dependent apoptosis.

AMPK and Akt are well known to negatively and positively regulate the mTOR pathway, respectively [39]. The mTOR signaling pathway is a regulator of several cellular processes, including proliferation, autophagy, and survival [40]. An intriguing finding based on our results is the induction of AMPK phosphorylation by HMDB as early as 6 h after the initiation of treatment accompanied with the suppression of phosphorylation of Akt, mTOR, Raptor, and S6K, suggesting that HMDB induces autophagy through inducing AMPK activation and subsequently blocking AKT and mTOR activation. The regulation of both Akt and mTOR signaling pathways by HMDB implicates another therapeutic benefit. The mechanism of resistance to mTORC1 inhibitors in endometrial cancer may result from a negative feedback loop emerging from receptor tyrosine kinase/PI3K/Akt/S6K signaling pathway [41,42]. This implies that losing the feedback inhibition of Akt/mTOR signaling resulted from the use of mTORC1 inhibitors may reduce their therapeutic capacities and lead to the following chemoresistance. Therefore, an agent with the ability to inhibit both mTORC1 and Akt, such as HMDB, could be beneficial for the treatment of cervical cancer.

**Figure 6:**
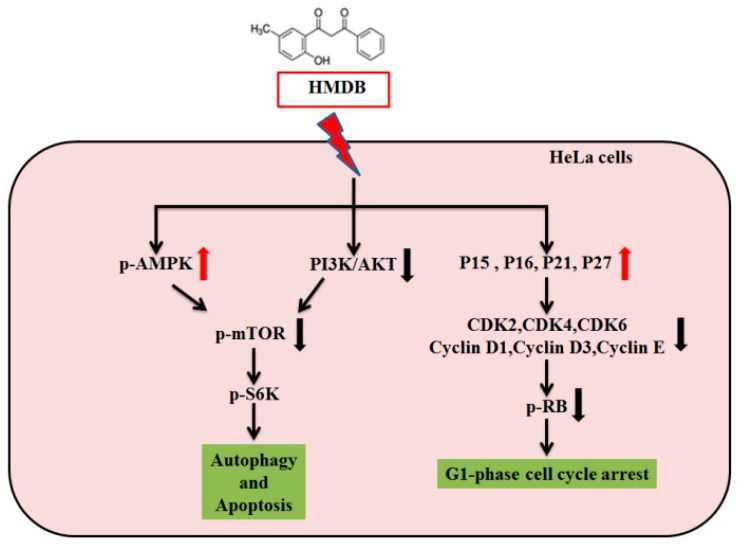
The proposed signal pathway activated by HMDB in HeLa cervical cancer cells. Red arrows represent increased protein expression by HMDB, while black arrows indicate a decrease.

To sum up, our results show a paralleled event that HMDB modulates several cellular signaling pathways and targets cell proliferation in human HeLa cervical cancer cells by involving in cell cycle arrest and induction of cell death via autophagy and apoptosis. The mechanism (Figure 6) by which HMDB induces G1 cell cycle arrest is due to the mediation of G1 cell cycle regulators, including the changes in the expression of D- and E-type cyclins, CDKs, CKIs, and Rb phosphorylation. We provide additional evidence that the autophagic cell death induced by HMDB is due to the activation of AMPK and the subsequent inhibition of mTORC1 activity. On the basis of these findings, we conclude that HMDB might potentially serve as a therapeutic agent for cervical cancer.

## 4. Materials and Methods

### 4.1. Chemicals and Reagents

1-(2-Hydroxy-5-methylphenyl)-3-pheyl-1,3-propanedione (HMDB) was purchased from Aldrich Chemical Co. (Milwaukee, WI, USA). The purity of the compound is >97% by high performance liquid chromatography (HPLC), and dissolved in dimethyl sulfoxide (DMSO). Propidium iodide, RNaseA, and 3-methyladenine (3MA) were available from Sigma-Aldrich (St. Louis, MO, USA). Antibodies against β-actin, Bcl-2, PCNA, and survivin were obtained from Santa Cruz Biotechnology (Santa Cruz, CA, USA). Antibodies against LC3A/B, phospho-AMPK (Thr172), AMPK, phospho-Akt (Ser473), Akt, phospho-mTOR (Ser2448), mTOR, phospho-Raptor (Ser792), Raptor, phospho-p70 S6K (Thr389), p70 S6K, and all G1 cell cycle regulators were from Cell Signaling Technology (Beverly, MA, USA). Antibodies against Beclin-1 and SQSTM1/p62 were purchased from GeneTex, Inc. (Irvine, CA, USA).

### 4.2. Cell Culture

HeLa cells were purchased from American Type Culture Collection (ATCC) and were cultured in Dulbecco’s minimal essential medium (DMEM) supplemented with 10% fetal calf serum (Gibco BRL, Grand Island, NY, USA), 100 units/mL of penicillin, and 100 μg/mL of streptomycin (Gibco BRL, Grand Island, NY, USA), and kept at 37 °C in a humidified atmosphere of 5% CO_2_ in air according to ATCC recommendations. For all results, the cells cultured showed no more than 20 passages.

### 4.3. MTT Assay

The cells were seeded into 96-well plates at 5 × 10^3^ cells/well for 24 h. HMDB was added with various concentrations, and the cells were incubated for the indicated times. After treatment, cell viability was assessed by the MTT (3-(4,5-dimethylthiazol-2-yl)-2,5-diphenyltetrazolium bromide) assay as follows: 20 μL of MTT solution (5 mg/mL, Sigma, St. Louis, MO, USA) was added to each well and incubated for 24 h at 37 °C. Then the supernatant was removed, and the MTT-formazan crystals formed by metabolically viable cells were dissolved in 200 μL of dimethyl sulfoxide (DMSO). The absorbance was monitored by a microplate reader at a wavelength of 570 nm.

### 4.4. Trypan Blue Dye Exclusion Assay

The cells were cultured in 6-well plates at the density of 5 × 10^4^ cells per well in triplicate. After 24 h, cells were treated with 40 μM HMDB for the indicated times. After treatment of HMDB, cells were washed with phosphate-buffered saline (PBS) and a solution of 0.125% trypsin, 0.05% ethylenediaminetetraacetic acid (EDTA) for 2 min, and then incubated with trypan blue solution (1:1 dilution) for 10 min. After staining, cells were transferred to a hemocytometer (Bright-line™; Hausser Scientific, Horsham, PA, USA) and counted by microscopy (Observer-A1; Carl Zeiss, Oberkochen, Germany). The cells stained with the trypan blue dye are defined as dead cells. The percentage of living cells represented the number of living cells divided by the total number of counted cells.

### 4.5. Cell Cycle Analysis

Cell cycle population was determined by flow cytometry as follows. After exposing to HMDB for 0, 6, 12, and 24 h, HeLa cells were washed twice with PBS, and then fixed in 70% ethanol for additional 2 h at −20 °C. Following fixation, cells were washed with PBS again, incubated with 1 mL of PBS containing 0.5 μg/mL RNase A and 0.5% Triton X-100 for 30 min at 37 °C. Then the cells were stained with 50 μg/mL propidium iodide (PI). The stained cells were estimated by a FACScan laser flow cytometer equipped with Cell Quest software (Becton Dickinson, San Jose, CA, USA).

### 4.6. Western Blotting

After HeLa cells were treated with the indicated concentration of HMDB for the indicated times, the cells were collected followed with PBS washing. Then, the cells were incubated in a lysis solution containing 50 mM Tris-HCl, pH 8.0, 5 mM EDTA, 150 mM NaCl, 0.5% NP-40, 0.5 mM phenylmethanesulfonyl fluoride, and 0.5 mM dithiothreitol for 30 min at 4 °C. Equal amounts of total proteins (50 μg) were subjected to sodium dodecyl sulfate-polyacrylamide gel electrophoresis (SDS-PAGE), followed by transferring to polyvinylidene difluoride (PVDF) membranes (Immobilon P, Millipore, Bedford, MA, USA) and incubating with primary antibodies. The membrane was further washed with PBST, incubated with corresponding peroxidase-conjugated goat anti-mouse or anti-rabbit secondary antibodies, and visualizing by enhanced chemiluminescence staining. The β-actin acts as an internal control to normalize protein loading. The density of the band on the blots was quantitated with a computerized densitometer (ImageQuant LAS4000 Digital System, GE Healthcare, Uppsala, Sweden).

### 4.7. RNA Extraction, cDNA Synthesis, and qRT-PCR

Total RNA from HMDB-treated HeLa cells was prepared using TRIzol reagent (Sigma), followed by the manufacturer’s instructions as below. Total RNA (5 μg) was applied to reverse transcription to generate cDNA by incubating the reaction mixture (25 μL) at 42 °C for 90 min containing Moloney murine leukemia virus (M-MLV) reverse transcriptase and oligo (dT) 18 primer. Then, real-time qPCR was executed in a 20 μL final volume for each primer (as below) using the Fastart Universal SYBR Green Master Mix (Roche, Indianapolis, IN, USA) and detected by an ABI 7000 sequence detection system. The primer sequences for p15, p16, p21, p27, and β-actin (β-actin is internal control) are as follows: β-actin (5′-AGTTGCGTTACACCCTTTCTTG-3′, 5′-CACCTTCACCGTTCCAGTTTT-3′), p15 (5′-GGCAGTCGATGCGTTCACTC-3′, 5′-CAGGGCTTCCAGAGAGTGTC-3′), p16 (5′-TTCCTGGACACGCTGGT-3′, 5′-CAATCGGGGATGTCTGAG-3′), p21 (5′-GCGACTGTGATGCGCTAAT-3′, 5′-TAGGGCTTCCTCTTGGAGAA-3′), p27 (5′-ATGTCAAACGTGCGAGTGTCTAA-3′, 5′-TTACGTTTGACGTCTTCTGAGG-3′). The PCR program is designed including the first reaction at 50 °C for 2 min and at 95 °C for 10 min, and then incubating for 40 thermal cycles between 95 °C for 15 s and 60 °C for 1 min. The relative cDNA expression for each sample was computerized using the formula 2^−∆∆*C*t^, where ∆∆*C*_t_  =  ∆*C*_t_(target gene) − ∆*C*_t_(β-actin gene), which represents the target cDNA expression normalized to β-actin cDNA levels.

### 4.8. Neutral Red Staining

Neutral red (Sigma-Aldrich, St. Louis, MO, USA) staining is designed to monitor the relative amounts of lysosomes or acidic vacuoles that are stained with the supravital dye neutral red. Briefly, following HMDB treatment, cells were washed and suspended in PBS. Next, cells were stained with neutral red (33 μg/mL) and visualized by phase contrast microscopy.

### 4.9. Monodansylcadaverine and Acridine Orange Staining

HeLa cells were treated with 40 μM HMDB for 0, 6, 12 and 24 h. After washing with fresh culture medium, the cells were stained with the autofluorescent dye containing 0.05 mM monodansylcadaverine (MDC) (Sigma-Aldrich, St. Louis, MO, USA) in PBS at 37 °C for 1 h, and then fixed with cold 4% paraformaldehyde for 15 min. For acridine orange staining, the cells were stained with 1 μg/mL acridine orange (AO) (Sigma-Aldrich, St. Louis, MO, USA) at 37 °C for 15 min, and then fixed with cold 4% paraformaldehyde for 15 min. The stained cells were washed with PBS three times and visualized under a contrast microscope.

### 4.10. Immunofluorescent Staining

HeLa cells were grown to approximately 70% confluence on a coverslip, and then incubated with to the indicated concentrations of HMDB for the indicated times. For immunostaining, the treated cells were washed with cold PBS, and fixed with 4% paraformaldehyde for 15 min at room temperature. Furthermore, the fixed cells were stained with rabbit anti-LC3 antibody for 24 h, rinsed with cold PBS, followed with incubation with goat anti-rabbit secondary antibody conjugated with fluorescein isothiocyanate or rhodamine (Sigma, St. Louis, MO, USA) for 30 min at room temperature. The immunolabeled cells were mounted on a glass slide with DAPI Dapi-Fluoromount-G (Southern Biotech, Birmingham, AL, USA) and observed using a fluorescence microscope (Carl Zeiss MicroImaging GmbH, Jena, Germany).

### 4.11. TUNEL Assay

DNA fragmentation analysis was performed by terminal deoxynucleotidyl transferase dUTP nick end labeling (TUNEL) assay using an in situ labeling cell death kit (Roche Applied Science, Indianapolis, IN, USA). As follows, cells were grown at a density of 5 × 10^4^ cells on a coverslip (25 mm size), followed with the incubation with 40 μM HMDB for the indicated times. The treated cells were washed with cold PBS, and rinsed with 4% paraformaldehyde for 15 min at 37 °C. Then the cells were incubated with a permeabilization solution containing 0.1% Triton X-100 in 0.1% sodium citrate for 5 min at 4 °C, and then applied to the TUNEL reaction buffer for 60 min at 37 °C in a humidified atmosphere in the dark. Afterward, the results were observed using a fluorescence microscope (Carl Zeiss MicroImaging GmbH, Jena, Germany).

### 4.12. Statistical Analysis

Quantitative data taken on the values from three or more replicates repeated experiments were representative as the mean value with the respective standard error of the mean (SE). One-way analysis of variance (ANOVA) using Tukey’s post hoc multiple comparisons was applied for multiple group comparison, and the analyzed values were considered statistically significant at *p* < 0.05.
